# Cysteine synthase: multiple structures of a key enzyme in cysteine synthesis and a potential drug target for Chagas disease and leishmaniasis

**DOI:** 10.1107/S2059798323003613

**Published:** 2023-05-19

**Authors:** Kate Sowerby, Stefanie Freitag-Pohl, Ana Milena Murillo, Ariel Mariano Silber, Ehmke Pohl

**Affiliations:** a Department of Chemistry, Durham University, Durham, United Kingdom; b Department of Parasitology, University of Sao Paulo, São Paulo, Brazil; University of Konstanz, Germany

**Keywords:** amino-acid metabolism, cysteine synthase, PLP-dependent enzymes, X-ray crystallography, Chagas disease, leishmaniasis, *Trypanosoma theileri*, *Trypanosoma cruzi*, *Leishmania infantum*

## Abstract

Chagas disease is a neglected tropical disease (NTD) caused by *Trypanosoma cruzi*, whilst leishmaniasis, which is caused by over 20 species of *Leishmania*, represents a group of NTDs endemic to most countries in the tropical and subtropical belt of the planet. These diseases remain a significant health problem both in endemic countries and globally. These parasites and other trypano­somatids, including *T. theileri*, a bovine pathogen, rely on cysteine biosynthesis for the production of trypanothione, which is essential for parasite survival in hosts. The *de novo* pathway of cysteine biosynthesis requires the conversion of *O*-acetyl-l-serine into l-cysteine, which is catalysed by cysteine synthase (CS). These enzymes present potential for drug development against *T. cruzi*, *Leishmania* spp. and *T. theileri*. To enable these possibilities, biochemical and crystallographic studies of CS from *T. cruzi* (*Tc*CS), *L. infantum* (*Li*CS) and *T. theileri* (*Tth*CS) were conducted. Crystal structures of the three enzymes were determined at resolutions of 1.80 Å for *Tc*CS, 1.75 Å for *Li*CS and 2.75 Å for *Tth*CS. These three homodimeric structures show the same overall fold and demonstrate that the active-site geometry is conserved, supporting a common reaction mechanism. Detailed structural analysis revealed reaction intermediates of the *de novo* pathway ranging from an apo structure of *Li*CS and holo structures of both *Tc*CS and *Tth*CS to the substrate-bound structure of *Tc*CS. These structures will allow exploration of the active site for the design of novel inhibitors. Additionally, unexpected binding sites discovered at the dimer interface represent new potential for the development of protein–protein inhibitors.

## Abstract 2

Biochemical and structural analyses of cysteine synthase, the key enzyme in cysteine biosynthesis, from the protozoan pathogens *Trypanosoma cruzi*, *T. theileri* and *Leishmania infantum* are presented. This enzyme is a potential drug target for neglected tropical diseases such as Chagas disease and leishmaniasis.

## 1. Introduction

Neglected tropical diseases (NTDs) are a wide-ranging group of diseases that affect over a billion people mainly in impoverished communities in tropical and subtropical areas around the globe. Among the 20 identified diseases which have been targeted by the World Health Organization for control and elimination are Chagas disease and leishmaniasis (World Health Organization, 2020 ▸).

Chagas disease, also known as American trypanosomiasis, is caused by *Trypanosoma cruzi* and affects 6–8 million people, mainly in the Americas; due to migration it has also become a global concern in non-endemic regions such as Canada, Europe, Japan and Australia (Lidani *et al.*, 2019 ▸). Leishmaniasis is caused by more than 15 species of trypanosomatids of the genus *Leishmania*. The disease is endemic in most tropical and subtropical regions of the world, and increasingly in the Mediterranean region (Burza *et al.*, 2018 ▸). A closely related trypanosomatid, *T. theileri*, is found globally and is capable of infecting livestock mammals, including sheep and cattle (Fentahun, 2020 ▸; Kelly *et al.*, 2017 ▸). Pathologies resulting from *T. theileri* infection have not been well investigated due to their presumed limited economic effects and are not entirely understood, but often occur due to co-infection with bovine leukaemia virus or *Theileria orientalis* (Brotánková *et al.*, 2022 ▸; Suganuma *et al.*, 2022 ▸). However, drugs against veterinary trypanosomatids such as *T. theileri* should be preventively developed under the scope of the One Health approach (Chimera *et al.*, 2021 ▸).

All three parasites belong to the trypanosomatid family and share a complex life cycle including an insect vector and a mammalian host. Chagas disease is mainly transmitted by a triatomine vector; when these obligate haematophagous insects are infected they spread *T. cruzi* through blood-feeding behaviour (Vieira *et al.*, 2018 ▸). *L. infantum* is primarily transmitted through phlebotomine sandflies (Alten *et al.*, 2016 ▸). *T. theileri* is mainly spread by Tabanidae flies, but also has the potential to be transmitted by dipteran vectors such as mosquitos and sandflies (Fentahun, 2020 ▸; Brotánková *et al.*, 2022 ▸). Due to several factors, including the variety of reservoirs and resistance to insecticides, vector control is insufficient to eradicate these parasites and hence emphasizes the need for affordable and effective drugs (Francis *et al.*, 2021 ▸).

Current treatments for Chagas disease are based on only two drugs, benznidazole and nifurtimox, which were introduced in the late 1960s. These drugs both aim to reduce the parasite load and to reduce transmission (Coura & Borges-Pereira, 2011 ▸). Both are prodrugs that are activated by nitroreductases to produce reactive metabolites, resulting in cellular toxicity and parasite death (Patterson & Wyllie, 2014 ▸; Boiani *et al.*, 2010 ▸). However, these treatments pose severe problems associated with their use, such as a low treatment-completion rate of only 65%, combined with 90% of patients experiencing severe side effects including headaches, fatigue, nausea and rashes (Jackson *et al.*, 2020 ▸). Furthermore, these drugs have limited efficacy during the chronic phase of the disease, when most patients are diagnosed (Müller Kratz *et al.*, 2018 ▸; de Oliveira *et al.*, 2021 ▸). For leishmaniasis, the main first-line treatments are pentavalent antimony derivatives. These drugs are severely toxic and are responsible for cardiotoxicity, pancreatitis and fever, among other side effects. In addition, their effective administration requires hospitalization. Other drugs such as liposomal amphotericin B and miltefosine are available as second-line treatments; however, these alternatives also have drawbacks such as expense and toxicity (Moore & Lockwood, 2010 ▸). In addition, increasing resistance to these drugs has been reported, limiting their efficacy (Masmoudi *et al.*, 2013 ▸). Current drug therapies for both Chagas disease and leishmaniasis are not satisfactory due to their limited efficacy, frequent side effects and long treatment regimens (Francisco *et al.*, 2020 ▸). Resistance to drug treatments for leishmaniasis is well known and has emerged over the past 30 years (Croft *et al.*, 2006 ▸). Further, the mode of action of these drugs is poorly understood, complicating any attempts to improve their efficacy. As a consequence, these neglected tropical diseases present great unmet medical challenges in the need for novel therapies.

**Figure 1:**
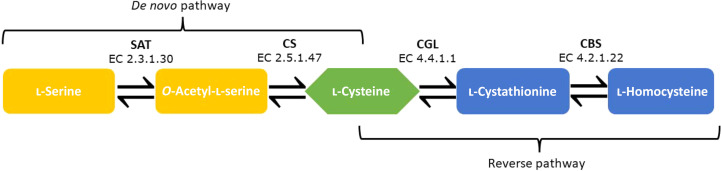
Schema of the *de novo* cysteine-synthesis and reverse transsulfuration pathways. The enzymes involved in these pathways are abbreviated as follows: SAT, serine acetyltransferase, CS, cysteine synthase, CGL, cysteine γ-lyase, CBS, cystathionine β-synthase.

Remarkably, the life cycles of all of these protozoan parasites rely on the ability of trypanosomatids to survive the oxidative imbalance imposed by the host response. The most essential low-molecular-weight thiol responsible for this is trypanothione, which consists of two molecules of glutathione linked by a spermidine bridge. Therefore, spermidine, glutathione and trypanothione are vital components of the detoxification pathways for reactive oxygen species in the parasite (Machado-Silva *et al.*, 2016 ▸). Importantly, the key metabolite providing thiol groups for the synthesis of glutathione and therefore trypanothione is the amino acid cysteine (Müller *et al.*, 2003 ▸). Over the last decade, mounting evidence has pointed to the importance of cysteine biosynthesis. In *T. cruzi*, cysteine synthesis can proceed through either the *de novo* pathway or the reverse transsulfuration pathway (Fig. 1 ▸). In the *de novo* pathway, l-serine is modified to *O*-acetyl-l-serine by serine acetyltransferase (SAT). *O*-Acetyl-l-serine (OAS) is then converted to l-cysteine in a reaction catalysed by cysteine synthase (CS; Nozaki *et al.*, 2001 ▸). Additionally, several groups have reported the formation of a cysteine synthase complex between SAT and CS (Kumaran *et al.*, 2009 ▸; Wang & Leyh, 2012 ▸).

CS has been studied in both eukaryotic and prokaryotic organisms. The crystal structures of eukaryotic CS (for example from *Arabidopsis thaliana* and *Entamoeba histolytica*) as well as prokaryotic CS (for example from *Salmonella enterica* serovar Typhimurium) have been described (Dharavath *et al.*, 2020 ▸; Bonner *et al.*, 2005 ▸; Burkhard *et al.*, 1998 ▸). These CS structures share the same overall fold, that of a highly conserved type II class of PLP-dependent enzyme (Schneider *et al.*, 2000 ▸). Additionally, the CS structures determined so far show that CS is typically a homodimer.

Despite the importance of CS and the *de novo* cysteine-synthesis pathway to parasite survival during redox imbalance in their hosts, the structure and mechanism of reaction of CS in *Trypanosoma* has not been studied in detail to date. As CS is not found in humans, it constitutes a potential drug target. Marciano and coworkers found that *T. cruzi* CS (*Tc*CS) was expressed in greater levels in amastigotes, suggesting that in the mammalian stages of infection cysteine synthesis relies heavily on the *de novo* pathway (Marciano *et al.*, 2012 ▸). Like other Trypanosomatida, *L. infantum* relies on the trypanothione redox system to survive the oxidative stress imposed by the mammalian immune system (Battista *et al.*, 2020 ▸). Although cysteine synthesis in *L. infantum* has not previously been studied in depth, analysis of genes encoding enzymes for cysteine synthesis in *L. major* has suggested that *L. infantum* also contains the two pathways of cysteine synthesis and thus will be capable of *de novo* cysteine synthesis (Williams *et al.*, 2009 ▸). Further, the structure of cysteine synthase from *L. donovani* has been determined using the putative *L. infantum* cysteine synthase gene sequence to identify the cysteine synthase, and thus *L. infantum* can be expected to have similar properties (Raj *et al.*, 2012 ▸). *T. theileri* has not been studied at all in this regard. The conservation of the genes in this pathway, not only in *T. cruzi*, *L. infantum* and *T. theileri* but also in other related protozoan parasites such as *L. major* and *E. histolytica*, clearly underlines the importance of these genes and the *de novo* pathway (Mori *et al.*, 2015 ▸; Fyfe *et al.*, 2012 ▸).

In this work, we cloned and produced recombinant CSs from the three trypanosomatids *T. cruzi*, *L. infantum* and *T. theileri*. This work is therefore of particular interest for the One Health approach. We show here that all three recombinant CSs are correctly folded and enzymatically active. Furthermore, high-resolution crystal structures of the three homologues are reported, revealing the apo, holo and substrate-bound states. The data presented here establish the first solid base for the rational design of inhibitors against these putative new targets.

## 2. Materials and methods

### 2.1. Sequence alignment of *T. cruzi*, *L. infantum* and *T. theileri* CS

Sequences for *T. cruzi* CS (*Tc*CS; UniProt code V5BWY7), *L. infantum* CS (*Li*CS; UniProt code A4ID39) and *T. theileri* CS (*Tth*CS; UniProt code A0A1X0P4R1) were retrieved and aligned using *Clustal Omeg*a (Madeira *et al.*, 2019 ▸). Sequence alignments were displayed with *ESPript* 3.0 (Robert & Gouet, 2014 ▸).

### 2.2. Protein expression, purification and crystallization

**Figure 2:**
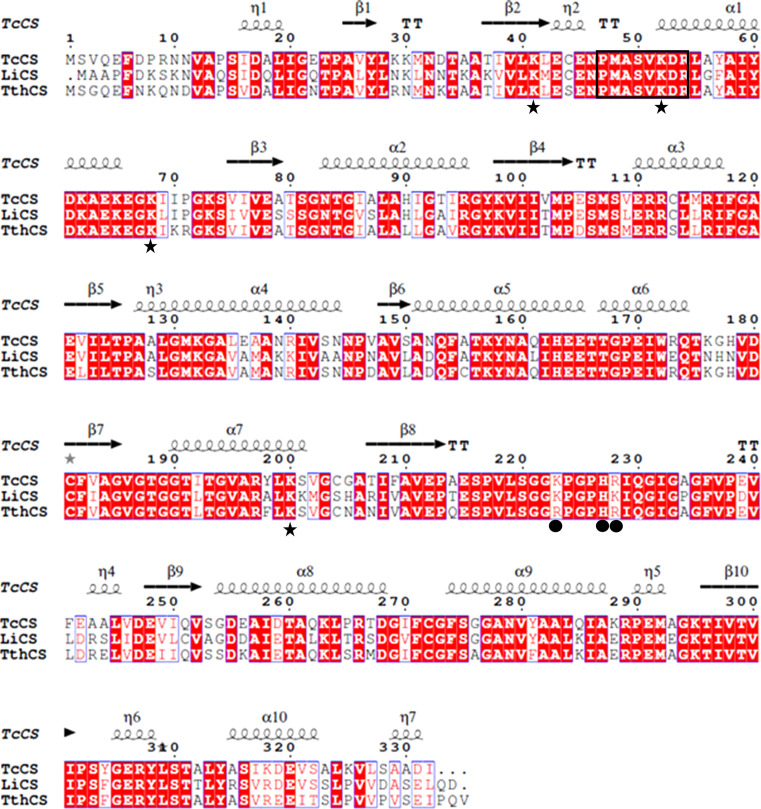
Multiple sequence alignment of the cysteine synthase sequences from *T. cruzi*, *L. infantum* and *T. theileri*. Identical residues are displayed in red boxes and similar residues in red text; similar or identical residues are framed in blue boxes. The secondary-structure annotation is based on the *Tc*CS structure presented here. Residues involved in catalytic activity are indicated by stars. SAT interaction residues are indicated by dots. The PLP box residues are outlined by a black box.

The three CSs from *T. cruzi*, *L. infantum* and *T. theileri* were codon-optimized for expression in *Escherichia coli* and purchased as synthetic genes for insertion into an expression plasmid by GenScript. The *Tc*CS coding sequence was cloned into the pJOE5751 expression vector (Wegerer *et al.*, 2008 ▸; Cornish *et al.*, 2022 ▸) between the BamHI and the BsrGI sites with an N-terminal His_6_ tag. The *Li*CS and *Tth*CS coding sequences were cloned into the pET-15b expression vector (Novagen) between the NdeI and XhoI sites with an N-terminal His_6_ tag. Numbered residues within this paper refer to *Tc*CS numbering as shown in Fig. 2 ▸.

### 2.3. Production and purification

The recombinant plasmids were transformed into *E. coli* BL21 (DE3) cells (NEB). Freshly transformed *E. coli* BL21 cells were grown in LB medium supplemented with 100 µg ml^−1^ ampicillin at 37°C to an OD_600_ of 0.4. The bacterial cultures containing pJOE5751_*Tc*CS were induced with 0.2% rhamnose (Sigma) for overexpression of *Tc*CS, whereas those containing pET-15b_*Li*CS and pET-15b_*Tth*CS were induced with 1 m*M* isopropyl β-d-1-thiogalactopyranoside (Sigma) for the production of *Li*CS and *Tth*CS, respectively. The cultures were incubated for a further 16 h at 37°C for *Tc*CS and at 30°C for *Li*CS and *Tth*CS. The cells were harvested by centrifugation at 4000*g* for 25 min at 4°C. The harvested cells were suspended in lysis buffer (50 m*M* Tris–HCl pH 7.5, 500 m*M* NaCl, 30 m*M* imidazole, 2 m*M* β-mercaptoethanol) and lysed by sonication (Bandelin SONOPULS HD2070) for 2 min at 50% power on ice. The cell lysates were centrifuged at 48 500*g* for 50 min at 4°C and the resulting supernatant was filtered through a 0.45 µm filter. The proteins were initially purified using a HisTrap HP nickel ion-affinity chromatography column (Cytiva) which was pre-equilibrated with lysis buffer. Elution buffer (50 m*M* Tris–HCl pH 7.5, 500 m*M* NaCl, 500 m*M* imidazole, 2 m*M* β-mercaptoethanol) was used to elute the protein over a 20 CV gradient. Protein fractions were analysed for homogeneity on SDS–PAGE and dialysed against HEPES buffer [20 m*M* HEPES pH 7.5, 300 m*M* NaCl, 10%(*w*/*v*) glycerol]. When required for crystallization and characterization, further purification was carried out by gel filtration using a HiLoad 16/600 Superdex 200 pg (Cytiva) and size-exclusion buffer (20 m*M* HEPES pH 7.5, 300 m*M* NaCl). Electrospray ionization time-of-flight mass spectrometry was performed to confirm that the protein was of the expected molecular weight.

### 2.4. Activity assays

To determine the enzymatic activity, the quantity of cysteine produced by the enzymes was determined by measuring the absorbance of Ruhemann’s purple produced by the reaction with cysteine (Friedman, 2004 ▸). The activity assay was formulated in a 96-well plate, in which all wells contained 150 n*M* protein, 50 m*M* Tris pH 7.5, 0.01 m*M* EDTA, 2 m*M* β-mercaptoethanol, 3 m*M* sodium sulfide and varied concentrations of the substrate *O*-acetylserine (OAS) from 0 to 10 m*M*. The reaction was started by the addition of protein. The plate was sealed with StarSeal Sealing Tape Polyolefin Film 100 (STARLAB) and incubated at 37°C for 30 min. The reaction was quenched by decreasing the pH of the solution with acetic acid. Cysteine production was quantified by the formation of Ruhemann’s purple. The amount of cysteine produced was determined by comparing the absorbance readings obtained with those from a calibration curve obtained using solutions of cysteine at defined concentrations as a standard.

### 2.5. Crystallization and data collection of *Tc*CS

Purified *Tc*CS was concentrated to 4 mg ml^−1^ as estimated using a DS-11+ Spectrophotometer (Denovix). A theoretical extinction coefficient of 20 650 *M* ^−1^ cm^−1^ at 280 nm was used to calculate the protein concentration (Gasteiger *et al.*, 2005 ▸). High-throughput crystallization trials were conducted with a range of commercially available 96-well crystallization screens (Molecular Dimensions) using a Mosquito system (SPT Labtech) in MCR crystallization plates. From these screens, promising conditions were obtained from several conditions containing ammonium sulfate. To optimize the initial crystallization conditions, hanging drops were prepared in 24-well plates (Molecular Dimensions) by mixing 1 µl protein solution with 1 µl reservoir solution and were equilibrated against 500 µl reservoir solution at 25°C. The best crystals of *Tc*CS were obtained using a reservoir solution consisting of 2.4 *M* ammonium sulfate, 0.1 *M* bis-Tris pH 6.0.

Crystals were mounted in cryoloops and cryoprotected with 25%(*v*/*v*) glycerol before flash-cooling them in liquid nitrogen (Teng, 1990 ▸). High-resolution diffraction data were collected at a wavelength of 0.9762 Å on the I03 beamline at Diamond Light Source (DLS) equipped with an EIGER2 XE 16M detector (Casanas *et al.*, 2016 ▸). Further experimental details are summarized in Table 1 ▸.

### 2.6. Crystallization and data collection of *Li*CS

Purified *Li*CS was concentrated to 12 mg ml^−1^ using a theoretical extinction coefficient of 15 930 *M* ^−1^ cm^−1^ at 280 nm to calculate the protein concentration (Gasteiger *et al.*, 2005 ▸). High-throughput crystallization trials were conducted as described previously, resulting in the formation of crystals in several conditions. Optimization of the initial crystals was conducted by setting up sitting drops in 24-well plates (Molecular Dimensions) by mixing 1 µl protein solution with 1 µl reservoir solution and were equilibrated against 500 µl reservoir solution at 25°C. The best crystals of *Li*CS were obtained using 0.2 *M* sodium chloride, 30%(*w*/*v*) PEG 3350, 0.1 *M* bis-Tris pH 5.8.

Crystals were mounted as described previously and high-resolution diffraction data were collected on the I24 beamline at DLS equipped with a PILATUS3 6M detector (Broennimann *et al.*, 2006 ▸). Data were collected at a wavelength of 0.9762 Å and two data sets were collected from a single crystal and merged. Statistics are shown in Table 1 ▸.

### 2.7. Crystallization and data collection of *Tth*CS

*Tth*CS was purified and concentrated to 12.7 mg ml^−1^ as calculated with a theoretical extinction coefficient of 15 930 *M* ^−1^ cm^−1^ at 280 nm (Gasteiger *et al.*, 2005 ▸). High-throughput crystallization trials were conducted as above using 96-well screens (Molecular Dimensions). Drops were set up with 1:1 and 2:1 protein:reservoir solutions in 200 and 300 nl drops, respectively. The best crystals of *Tth*CS from these screens were obtained with a reservoir solution consisting of 2.7 *M* ammonium sulfate, 0.1 *M* sodium cacodylate pH 6.5. Crystals were mounted as described previously and high-resolution diffraction data were collected on the I24 beamline at DLS using a PILATUS3 6M detector. Data were collected at a wavelength of 0.9762 Å. Further statistics are shown in Table 1 ▸.

### 2.8. Data processing and structure solution

Diffraction data were processed using *xia*2/*DIALS* (Winter, 2010 ▸). The *Tc*CS structure was determined by molecular replacement using *L. major* CS (PDB entry 4air; Fyfe *et al.*, 2012 ▸), which shares 78% sequence identity with *Tc*CS, as the search model. Molecular replacement was performed using *Phaser* in *CCP*4*i*2 (McCoy *et al.*, 2007 ▸; Potterton *et al.*, 2018 ▸) to search for four molecules in the asymmetric unit based on the Matthews coefficient (Matthews, 1968 ▸). Due to pseudosymmetry (β is close to 90°) within the crystal, initial structure solution in space group *P*2_1_2_1_2_1_ failed. The data were reprocessed in space group *P*2_1_ and molecular replacement was successful. *Parrot* (Cowtan, 2010 ▸) was used to improve the electron density of the map; *Buccaneer* (Cowtan, 2006 ▸) was then used to build initial protein side chains. Refinement was performed using noncrystallographic symmetry restraints in *REFMAC* (Murshudov *et al.*, 2011 ▸; Headd *et al.*, 2014 ▸; Usón *et al.*, 1999 ▸) and was monitored using *R*_free_ (Brünger, 1992 ▸). *Coot *was utilized for manual model building with water and ligand incorporation (Emsley *et al.*, 2010 ▸).

The *Li*CS and *Tth*CS structures were determined by molecular replacement using *Tc*CS as the search model. For both structures, molecular replacement was performed using *Phaser* (McCoy *et al.*, 2007 ▸) to search for two molecules in the asymmetric unit based on the Matthews coefficient. Model building and refinement were completed as described above. Images were generated using *CCP*4*mg* (McNicholas *et al.*, 2011 ▸) and *PyMOL* (version 1.8; Schrödinger). Least-squares superpositions were performed using *CCP*4*mg*. Further crystallographic statistics for all structures are summarized in Table 1 ▸.

## 3. Results

### 3.1. Bioinformatic analysis of cysteine synthases

To understand the similarities and differences between the three cysteine synthases, a bioinformatic analysis of the protein sequences was undertaken. Sequence alignment of the three CS sequences from *T. cruzi*, *L. infantum* and *T. theileri* reveals proteins of a similar predicted size with high sequence similarity between them, as shown in Fig. 2 ▸. The sequence identity between *Li*CS and *Tc*CS is 72% and that between *Tth*CS and *Tc*CS is 80%, indicating that the enzymes are closely related. Further sequence analysis revealed that all three CS enzymes contain the four canonical lysine residues required for catalytic activity (Lys41, Lys52, Lys68 and Lys200) and the highly conserved consensus sequence for the PLP cofactor motif (P*XX*SVKDR) between Pro47 and Arg54. These lysine residues are known to have catalytic importance in orthologues from other organisms, including *T. rangeli* (Romero *et al.*, 2014 ▸). Lys52 is the putative cofactor-binding residue that forms a Schiff base with the PLP cofactor.

Certain orthologues of CS, such as the enzyme from *E. coli*, have been shown to form a stable complex with SAT, the first enzyme in the *de novo* synthesis pathway (Wang & Leyh, 2012 ▸). Lys223, His227 and Lys228 have previously been identified to be involved in SAT binding (Romero *et al.*, 2015 ▸). In *Li*CS, sequence analysis shows that all three residues are conserved. In *Tc*CS, only Lys223 and His227 are conserved, with Lys228 having changed to Arg228. In *Tth*CS, the only residue of this triad that is conserved is His227; both lysine residues have changed to arginines, with arginine also being positively charged and of a similar size. This suggests that all three enzymes are capable of the same interaction with SAT.

In conclusion, bioinformatic analysis of the *Tc*CS, *Li*CS and *Tth*CS sequences shows that both the canonical residues for catalytic activity and the binding sequence for the PLP cofactor are conserved, suggesting that the enzymes are able to bind both the PLP cofactor and the intermediates of the reaction pathway. Further, these enzymes are likely to interact directly with SAT in the catalytic cascade.

### 3.2. Characterization and biochemical activities of cysteine synthases

In order to ensure that all three proteins were of the expected size, mass spectrometry was performed. All three proteins are of the expected theoretical size; however, *Tc*CS also shows an additional mass of 232 Da indicating that PLP is bound to lysine to form a Schiff base. Further characterization was performed by gel filtration. This indicated that all three proteins are dimers in solution (data not shown).

**Figure 3:**
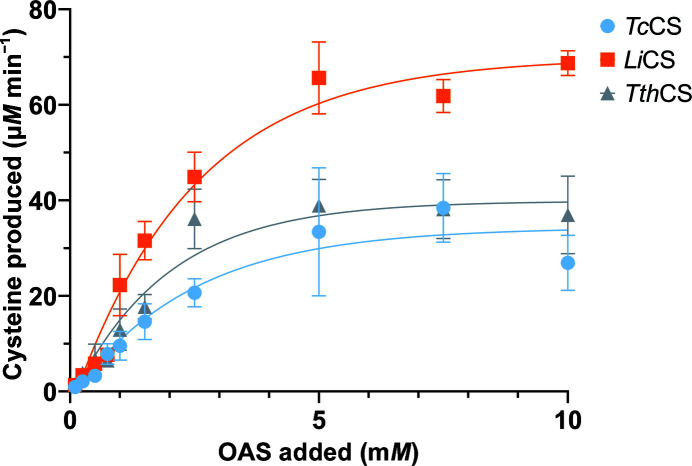
Enzymatic activities of *Tc*CS (blue), *Li*CS (orange) and *Tth*CS (grey). The enzymatic activity is determined by the amount of cysteine produced with increasing OAS concentration. In general *n* = 6, and a minimum of four data points were used per measurement.

To demonstrate that all three cloned genes encode active enzymes, the recombinant proteins were used to measure cysteine production using OAS and H_2_S as substrates. Fig. 3 ▸ shows that cysteine production is dependent on the amount of OAS. All CS proteins are capable of converting OAS into cysteine, releasing acetate, in an OAS-dependent manner. Under the conditions of the experiment, *Li*CS exhibits approximately double the *V*_max_ compared with the other two enzymes, indicating a faster reaction. The Michaelis constants *K*_m_ are all in the same range for the three enzymes, indicating similar substrate affinities.

### 3.3. Structure of *L. infantum* cysteine synthase

**Figure 4:**
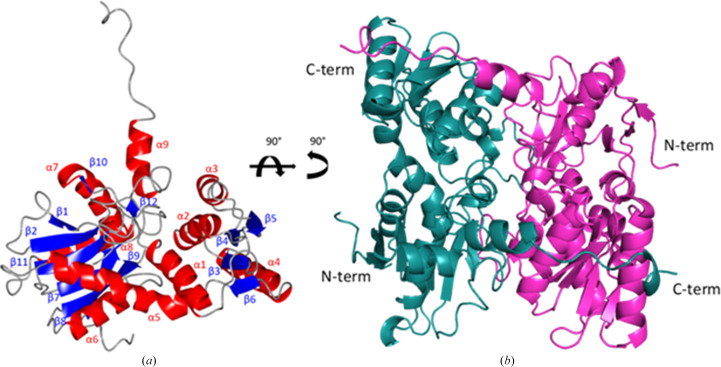
(*a*) Ribbon diagram of the *L. infantum* cysteine synthase monomer with secondary-structure elements labelled. (*b*) Ribbon diagram of the *Li*CS dimer. Chain *A* is shown in pink with chain *B* in teal. The monomer shown in (*a*) is rotated 90° around two axes to form the orientation displayed in (*b*).

To study the structure of *Li*CS, protein crystals were produced and the structure was solved at 1.80 Å resolution (Table 1 ▸). The protein crystallizes with one homodimer with almost identical monomer structures in the asymmetric unit (Fig. 4 ▸). The r.m.s.d. of C^α^ atoms between the chains was 0.72 Å (Supplementary Table S1).

Each *Li*CS monomer adopts a compact structure composed of nine α-helices and 12 β-sheets with an extended C-terminal tail. With the exception of β1, all β-sheets run parallel. The C-terminal tail of each chain is formed by a flexible region that stretches across to the partner subunit of each dimer, forming an extensive interface. Further analysis of the homodimer with *PISA* shows that *Li*CS has a large dimer interface involving 27.0% of chain *A* residues and 26.6% of chain *B* residues (Krissinel & Henrick, 2007 ▸). This is an extensive interface with a buried surface of 3246 A^2^. The active site of *Li*CS is found at the centre of each subunit between α1 and α5. Although the purified protein was yellow in colour, suggesting the presence of the cofactor PLP, no electron density for the cofactor was observed. It is likely that the PLP was lost during crystallization. As no PLP was identified at the active-site lysine of *Li*CS, this conformation corresponds to the apo form of the enzyme.

### 3.4. Structure of *T. theileri* cysteine synthase

**Figure 5:**
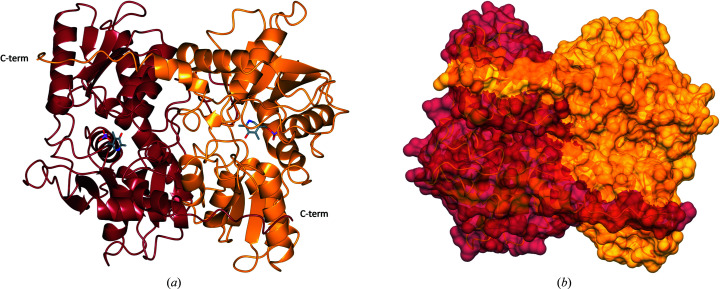
(*a*) Ribbon diagram of the *T. theileri* cysteine synthase dimer. The *Tth*CS dimer is shown in a similar orientation to the *Li*CS dimer in Fig. 4 ▸. Chain *A* is shown in yellow and chain *B* is in orange. (*b*) Dimer interface of *Tth*CS depicted in surface representation. Chain *A* is shown in yellow with residues involved in the dimer interface shown in gold. Chain *B* is in orange with residues involved in the dimer interface shown in brown.

To explore the mechanism of cysteine synthesis in further atomic detail, the structure of *Tth*CS was solved at 2.75 Å resolution (Table 1 ▸). The *Tth*CS structure, shown in Fig. 5 ▸, crystallized with one dimer per asymmetric unit and adopts the same overall fold as the apo *Li*CS structure. The two monomers have almost identical conformations, with an r.m.s.d. of 0.74 Å (Supplementary Table S1). The crystal structure of the *Tth*CS dimer also reveals an extensive dimer interface with buried surface areas of 3053 Å^2^ for chain *A* and 3099 Å^2^ for chain *B*. The observation that 25% of the protein is involved in dimer interactions clearly indicates that the enzyme is a functional homodimer (Krissinel & Henrick, 2007 ▸).

### 3.5. PLP is covalently bound to Lys52 in the active site of *Tth*CS

**Figure 6:**
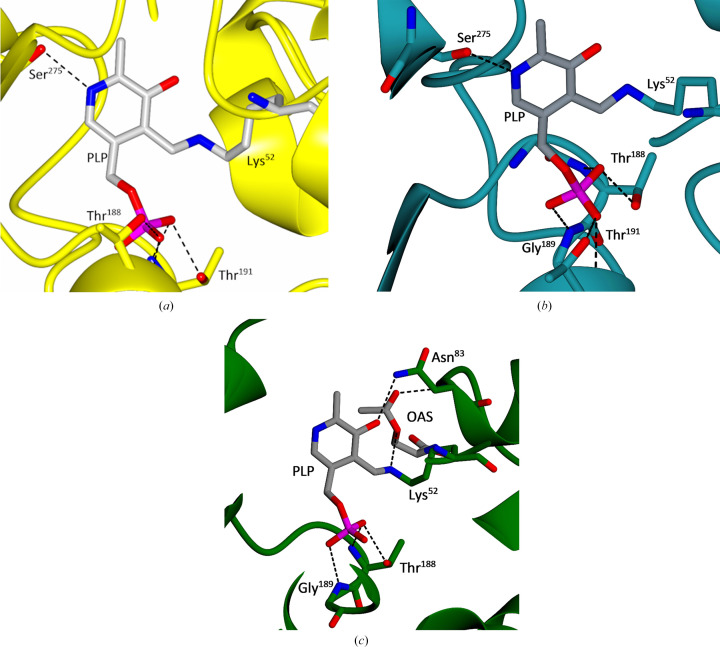
(*a*) Close-up of the active site in chain *A* of *Tth*CS showing the protein chain in ribbon representation and the PLP cofactor and the side chains of Lys52, Thr188, Thr191 and Ser275 in stick representation, with N atoms in blue, O atoms in red, C atoms in grey and P atoms in magenta. Key hydrogen bonds are indicated by dashed lines. Residue numbering refers to the *Tc*CS sequence in Fig. 2 ▸. (*b*) Close-up of the active site of *Tc*CS. PLP, Lys52, Thr188, Gly189, Thr191 and Ser275 are shown in stick representation using the same colour coding for atoms as before. Dashed lines indicate key hydrogen bonds. (*c*) Close-up showing the active site of *Tc*CS chain *A*. PLP, OAS, Lys52, Thr188, Gly189 and Asn83 are shown in stick representation using the same colour-coding for atoms as before. Dashed lines indicate hydrogen bonds.

In the active site of cysteine synthase, the PLP cofactor is covalently bound to the active-site lysine. In each chain of *Tth*CS continuous electron density was found which fits the structure of the cofactor. Therefore, in this structure the covalently bound state of PLP has been captured (Fig. 6 ▸ and Supplementary Fig. S1). Both subunits of the dimer have PLP covalently bound at Lys52, forming a Schiff base which establishes hydrogen bonds to Thr188, Thr191 and Ser275. As seen in the multiple sequence alignment of the proteins (Fig. 2 ▸), the affected residues are conserved.

The PLP is located between α1 and α5. The binding of PLP confirms the involvement of the residues predicted by bio­informatic analysis. These conserved residues, especially Lys52 which is involved in PLP binding through a covalent bond, are shown in Fig. 6 ▸. Thr188, Thr191 and Ser275 all form conserved hydrogen bonds to PLP.

### 3.6. Structure of *T. cruzi* cysteine synthase

The structure of *Tc*CS was determined at 1.80 Å resolution (Table 1 ▸). The protein crystallized with two independent dimers in the asymmetric unit, resulting in pseudosymmetry. The initial diffraction data suggested an orthorhombic cell in space group *P*2_1_2_1_2_1_ with one protein dimer in the asymmetric unit. However, further analysis revealed this to be pseudo-symmetry with one unit-cell angle very close to 90°. The data were consequently processed in the monoclinic space group *P*2_1_ with two independent dimers in the asymmetric unit. Gel-filtration experiments conducted with the protein in solution, in addition to the support of the crystal structure, show that *Tc*CS is functional as a homodimer.

**Figure 7:**
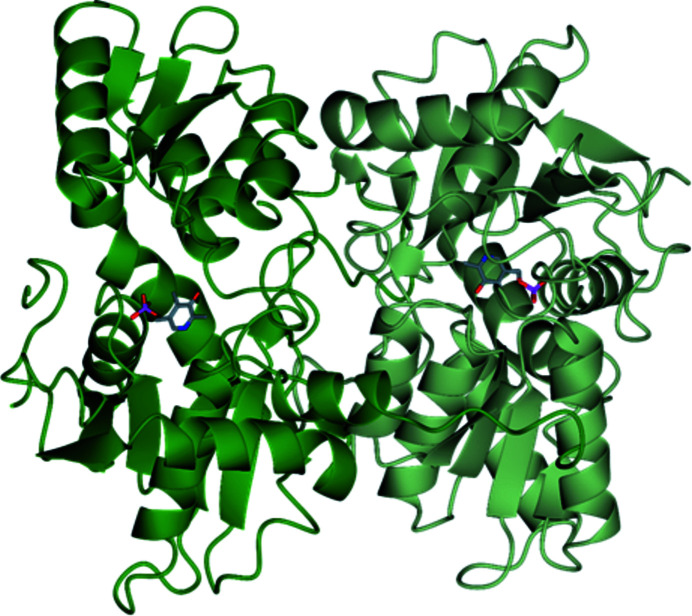
Ribbon diagram of the structure of *T. cruzi* cysteine synthase in a similar orientation to *Li*CS (Fig. 4 ▸) and *Tth*CS (Fig. 5 ▸). Chain *A* is coloured dark green and chain *B* is coloured light green. PLP is shown in stick representation.

The overall fold of all four protein chains is basically identical, with r.m.s.d.s ranging from 0.30 to 0.83 Å (Supplementary Table S1). The *Tc*CS model includes 344 residues per chain; however, the density for residues begins at Val3 so previous residues were not included. Residues 221–230 in chain *A*, 315–325 in chain *B* and 319–325 in chain *D* were poorly ordered and thus were not placed in electron density. As expected, given the high level of sequence identity, *Tc*CS shares a similar fold with both *Li*CS and *Tth*CS, with the exception of the C-terminus. The C-terminal tail of each chain is formed by a flexible region that stretches across to the partner subunit of each dimer, as shown in Fig. 7 ▸.

### 3.7. Dimer interface of *Tc*CS

**Figure 8:**
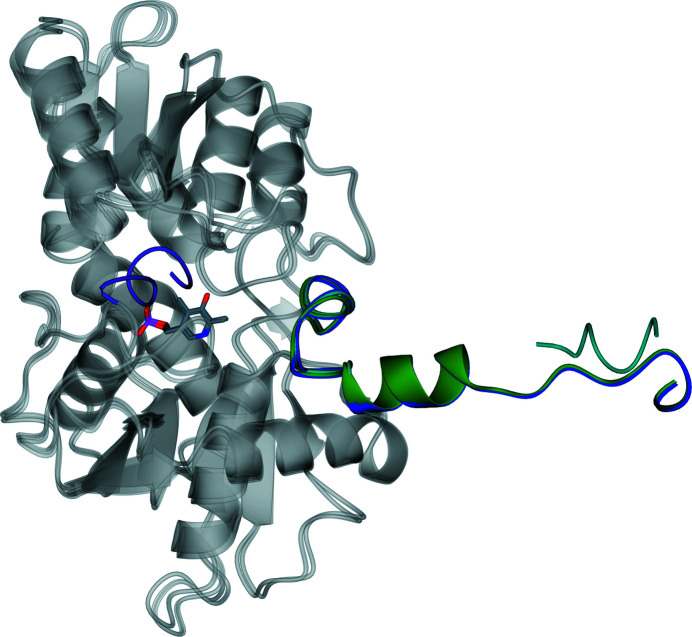
Ribbon diagram of superposed *Tc*CS chains. The C-terminal tail of chain *A* is in dark green, that of chain *B* is in purple, that of chain *C* is in blue and that of chain *D* is in teal. All tails are represented as ribbons.

Analysis with *PISA* (Krissinel & Henrick, 2007 ▸) shows that *Tc*CS forms an extensive interface in both crystallographically independent dimers. Chain *A* has a buried surface of 2862 A^2^, whilst chain *B* has a buried surface of 2774 A^2^. In contrast, chain *C* has a buried surface of 3037 A^2^ and chain *D* has a buried surface of 2981 A^2^. In both subunits the key inter­actions of the dimer are formed by the C-terminal tails; these flexible regions are varied, as shown in Fig. 8 ▸. Within the two dimers of *Tc*CS the *AB* dimer of *Tc*CS has an interface of 73 residues (23.4% of the total residues) for chain *A* and 71 residues (22.5% of the total residues) for chain *B*. There is a greater amount of interface in the *CD* dimer, which is formed from 82 residues (24.8% of the total residues) of chain *C* and 78 residues (24.3% of the total residues) of chain *D*. The C-terminal tails of chains *A*, *C* and *D* extend to the partner subunit (shown in Fig. 8 ▸); however, the C-terminal tail of chain *B* varies. Initially, the C-terminus of chain *B* starts out like the C-terminal tails in the other chains; however, residues 315–325 of chain *B* are disordered and residue 326 to the end of the C-terminus are found near the active site (Fig. 8 ▸). These two dimers show that there is significant flexibility of the C-terminus; however, given the location of the active sites this is unlikely to be relevant to catalysis and rather due to crystallization.

**Figure 9:**
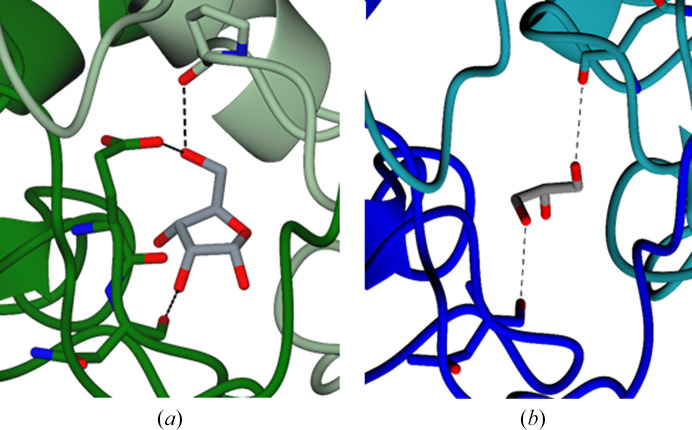
(*a*) Ribbon diagram of the dimer interface of chains *A* and *B* in *Tc*CS. Chain *A* is in dark green and chain *B* is in light green. Residues forming hydrogen bonds and ribose are shown in stick representation, with hydrogen bonds displayed as black dashed lines. (*b*) Ribbon diagram of the dimer interface of chains *C* and *D* in *Tc*CS. Chain *C* is in blue and chain *D* is in dark teal. Residues forming hydrogen bonds and glycerol are shown in stick representation, with hydrogen bonds displayed as black dashed lines.

Small molecules were found to be bound at the dimer interface of both *Tc*CS dimers. Based on the unbiased electron density (Fig. 9 ▸ and Supplementary Fig. S2), ribose was identified in the *AB* interface, whereas glycerol was found in the *CD* dimer (Fig. 9 ▸ and Supplementary Fig. S3). The presence of these two molecules suggests that a hydrophilic pocket is formed at the dimer interface.

### 3.8. PLP is covalently bound to Lys52 in the active site of *Tc*CS

In the active site of each *Tc*CS subunit, one molecule of PLP was found to be covalently bound to Lys52, as shown in Fig. 6 ▸(*b*). Additionally, Thr188, Gly189, Thr191 and Ser275 all form hydrogen bonds to the ligand. These residues are highly conserved throughout the CS sequences, showing the importance of these residues to the binding of the cofactor to allow the reaction to proceed. The PLP-binding site is located in between α1 and α5, as previously seen in other structures. The residues involved in the binding of PLP are the same residues that were identified previously during bioinformatic analysis.

### 3.9. OAS is present in the active site of *Tc*CS

In the active site of subunit *A*, additional density to the PLP was present (shown in Supplementary Fig. S5). This density is fitted by *O*-acetyl-l-serine, an essential substrate of the cysteine-synthesis reaction. The capture of this intermediate aligns with the canonical reaction mechanism for *Tc*CS shown in Fig. 6 ▸(*c*). The addition of OAS to the active site is representative of step 2 in Supplementary Fig. S6. Lys52 and Asn83 both form hydrogen bonds to OAS. The reactive N atom of the OAS is 5.7 Å from the O atom of PLP that together form the Schiff base.

## 4. Discussion

There is increasing evidence for the importance of the *de novo* cysteine-synthesis pathway for the survival of the pathogen and therefore pathogenesis in the mammalian host. The reliance of trypanosomatids such as *T. cruzi* and *L. infantum* on metabolites based on cysteine to survive the oxidative bursts imposed by the immune system of the host upon invasion presents an attractive drug target (Battista *et al.*, 2020 ▸). This survival is achieved through redox control using trypanothione and monoglutathionylspermidine (Krauth-Siegel & Comini, 2008 ▸); cysteine is essential for the creation of both molecules. Cysteine synthase is a critical enzyme in the *de novo* cysteine-synthesis pathway and hence presents a putative novel drug target for the treatment of Chagas disease and leishmaniasis.

A cysteine-synthesis reaction via CS has been described for orthologues (Romero *et al.*, 2014 ▸; Schnell *et al.*, 2015 ▸). Due to their similarity in sequence, the enzyme reaction in trypanosomatids is likely to proceed through the same mechanism (Supplementary Fig. S6). The PLP and the lysine residue at the active site form a Schiff base, also known as an internal aldimine. When OAS is present together with PLP, it forms an external aldimine. Through β-elimination of the acetate and deprotonation of the C^α^ atom of OAS, the external aldimine is converted to an α-aminoacrylate intermediate. This intermediate undergoes nucleophilic attack by sulfide, reforming the external aldimine. Through reprotonation of the C^α^ atom, l-cysteine is formed. The PLP and lysine bond reforms and the cycle continues.

### 4.1. Comparison of the activities of *Tc*CS, *Tth*CS and *Li*CS

As shown in functional assays, all three enzymes are highly active and capable of catalysing the biosynthesis of cysteine. At low concentrations of OAS (0–1 m*M*) the activities of all three enzymes are very similar, with all of them producing ∼0.5 m*M* cysteine at 1 m*M* OAS. The activities of *Tc*CS and *Tth*CS remain comparable, reaching a plateau at all OAS concentrations tested. This indicates that these enzymes reach maximum production at 1.2 m*M* cysteine. These similarities in activity are expected due to the high sequence identity, with an expected similar reliance on cysteine synthase in the *de novo* pathway. In comparison, under the conditions of the assay *Li*CS produces significantly higher concentrations of cysteine than *Tc*CS and *Tth*CS, reaching 2 m*M* cysteine before plateauing at 5 m*M* OAS. This is nearly double the production by *Tc*CS and *Tth*CS, which indicates that under the conditions of the assay *L*iCS is a more active enzyme when higher OAS concentrations are used.

As shown in Table 2 ▸, the *K*_m_ for OAS for all three proteins is similar. When compared with *Arabidopsis thaliana* CS, with a *K*_m_ of 1.4 m*M*, the plant CS shows a greater affinity for OAS than *Li*CS, *Tth*CS and *Tc*CS (Bonner *et al.*, 2005 ▸). In contrast, when compared with a bacterial CS such as that from *Trichomonas vaginalis*, which has a *K*_m_ of 39.5 m*M*, or the CS from the archaeon *Aeropyrum pernix*, which has a *K*_m_ of 21 m*M*, all three trypanosomatid proteins have a greater affinity for OAS (Westrop *et al.*, 2006 ▸; Mino & Ishikawa, 2003 ▸).

### 4.2. Comparison of the *Tc*CS, *Li*CS and *Tth*CS structures

**Figure 10:**
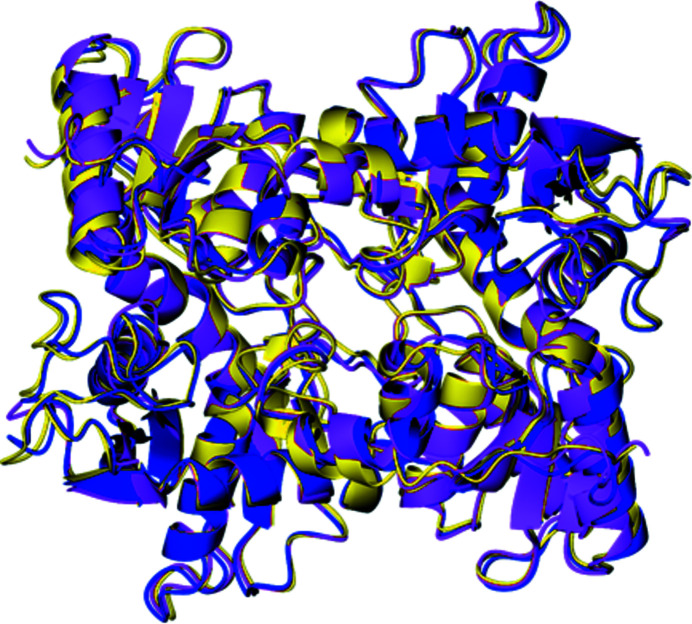
Ribbon diagram showing a least-squares superposition of the cysteine synthase structures. *Tc*CS is shown in dark blue, *Li*CS is shown in pink and *Tth*CS is shown in yellow.

The overall folds of all three CS structures are similar, as can be seen in the least-squares superposition (Fig. 10 ▸). The r.m.s.d.s are in the range 0.6–1.1 Å (Supplementary Table S1). Based on all experiments, the high sequence identity between the proteins, gel-filtration experiments and crystal structures, it can be concluded that all three proteins are functional dimers in solution. This is consistent with the sequence similarity between the proteins and previous CS structures. These key similarities can particularly be seen at the active site. This suggests that the reaction mechanism for all three enzymes is the same, implying that inhibition affecting the active site would be effective for all three CS enzymes. Furthermore, individual findings from each enzyme can be transferred between structures to provide a more comprehensive understanding of their molecular mechanism.

**Figure 11:**
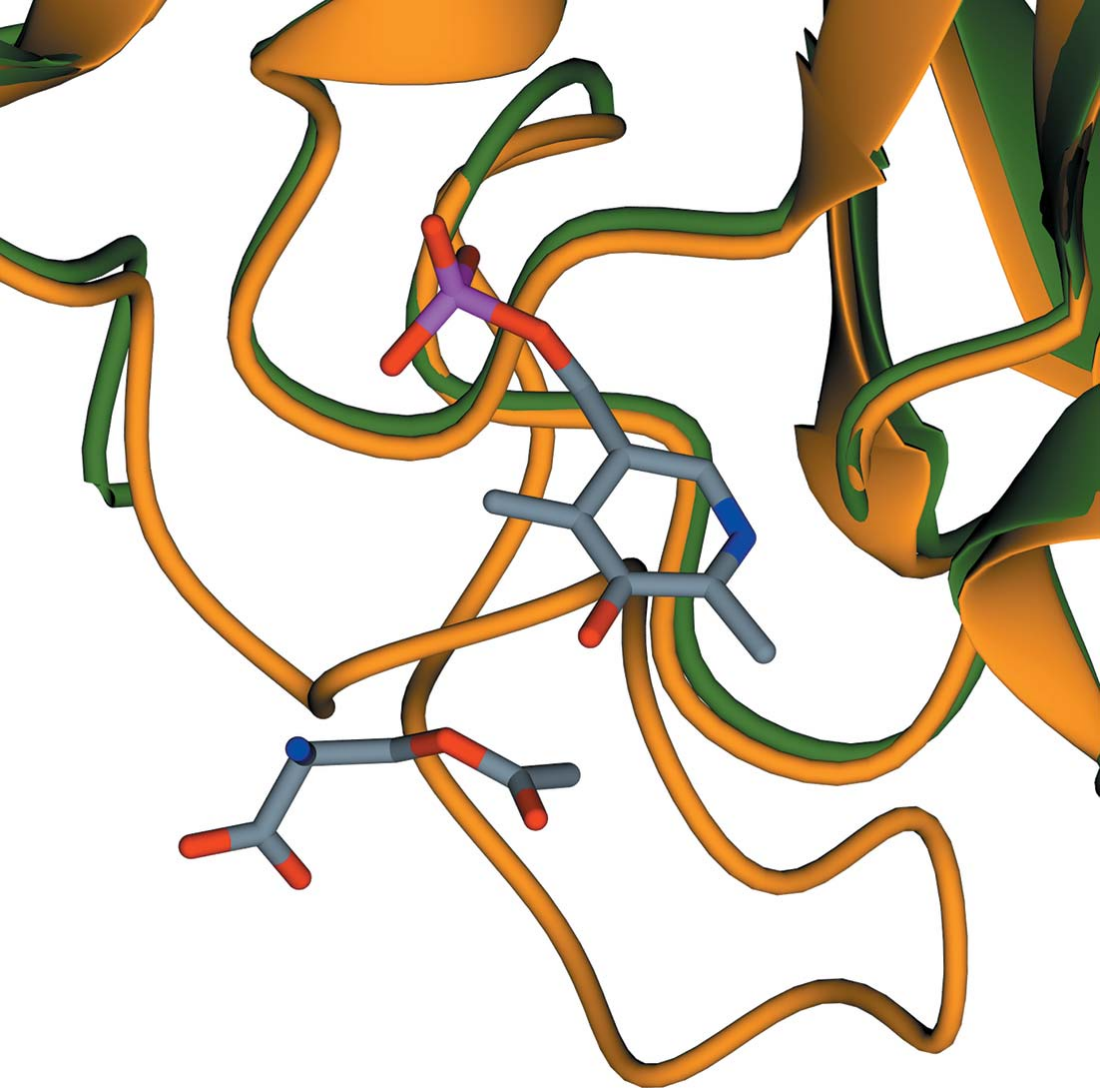
Ribbon diagram showing the active site of *Tc*CS of chain *A* superposed with chain *B* showing the closed form. Chain *A* is shown in dark green and chain *B* is in orange. PLP and OAS are shown in stick representation; atoms are coloured as before.

*Tc*CS and *Tth*CS both have PLP bound in the active site and therefore represent holo forms of the enzyme. PLP and OAS were not added to either protein during expression or purification and were likely to have been scavenged from the *E. coli* cells in which the protein was expressed. In these two structures a Schiff base is formed by a lysine (Lys52 in *Tc*CS and *Tth*CS) with PLP. This PLP allows catalysis of the conversion of OAS to l-cysteine and therefore the function of the enzyme. The residues involved in binding to PLP and OAS are listed in Table 3 ▸. In contrast, *Li*CS forms an apo CS structure as no PLP is bound in the active site. The *Li*CS structure and chain *A* of *Tc*CS are in the open form, whereas chains *B*, *C* and *D* of *Tc*CS and *Tth*CS are in the closed form (Fig. 11 ▸). The loop that covers the active site in *Tc*CS (residues 221–237) and *Tth*CS (residues 221–233) is thus not visible in the electron density of the *Li*CS structure due to the flexibility of this loop.

All three CS structures represent different stages along the cysteine-synthesis reaction pathway. The apo form determined for *Li*CS shows the protein in the absence of the cofactor. The holo form of *Tth*CS confirms the residues involved in binding PLP. The presence of OAS in the active site of *Tc*CS shows the structural changes that occur in the presence of reaction intermediates. This also reveals the active-site residues that interact with OAS. The position of OAS within the active site of *Tc*CS shows significant variability in *Tc*CS, which is consistent with results from other structures, for example that of *O*-acetylserine sulfhydrylase from *Haemophilus influenzae* in complex with OAS (PDB entry 5dbe; A. K. Singh, A. Kaushik, M. K. Ekka & S. Kumaran, unpublished work). The placement of OAS in this position is consistent with unbiased electron density; however, to complete the next step of the reaction the PLP needs to react with the amino group of the OAS to form the intermediate Schiff base. This step will require a re­arrangement of the lysine side chain as well as of the substrate. Taken together, the structures presented here provide valuable insight into the *de novo* cysteine-synthesis mechanism of trypanosomatid parasites.

### 4.3. Molecules at the dimer interface of *Tc*CS

In *Tc*CS, ribose and glycerol were found in hydrophilic pockets at the dimer interfaces of the *AB* and *CD* dimers, respectively. No ribose was added to the growth medium, suggesting that this molecule was scavenged from the *E. coli* cells during growth. Ribose is an abundant sugar with great cellular importance and therefore would be present in the cell during protein production. Glycerol was present in the buffer after purification and was also used in the crystallization conditions and as the cryoprotectant. Both are hydrophilic molecules and the presence of two different molecules demonstrate a conserved hydrophilic pocket formed by the dimer interface. This site could present an allosteric binding site at the dimer interface; however, further biochemical investigation will be required. Additionally, this location may represent a promising starting point for the development of a protein–protein interaction inhibitor for the interruption of dimer formation.

### 4.4. Flexible loop around the active site of *Tc*CS

There is considerable conformational flexibility around the active site in the *AB* dimer of *Tc*CS. In the absence of OAS in the active site, a loop formed of residues 221–237 covers the active site, as shown in Fig. 11 ▸. In the presence of OAS this loop shows increased flexibility. The absence of this loop over the active site allows substrates such as hydrogen sulfide to interact with the OAS and PLP. This shows that cysteine synthase has both an open and a closed form. The contrast between the two structures shows the structural changes that can occur during different stages of the reaction cycle.

Further evidence that these CS structures form a basis for drug discovery can be seen in previous studies using similar CS structures, such as work with CS from *S. enterica* serovar Typhimurium (*St*CS) to find an inhibitor against this bacterial pathogen (Annunziato *et al.*, 2021 ▸). Despite a sequence identity of only 47%, *St*CS has an r.m.s.d. of 1.2 Å over 283 residues when superposed with *Tc*CS. This difference in sequence identity is to be expected as *St*CS is a bacterial protein rather than a trypanosomatid protein. The surprisingly high r.m.s.d. is likely to be due to the highly conserved fold; however, the inhibitor determined in the *St*CS structure fits to the active site of *Tc*CS with the PLP matching and the inhibitor located in the same place as OAS.

## 5. Conclusions

Cysteine biosynthesis is critical to trypanosomatid survival upon host invasion due to the dependence of trypanothione on the availability of cysteine (Battista *et al.*, 2020 ▸). In trypanosomatids, cysteine biosynthesis proceeds through the *de novo* and reverse synthesis pathways. The *de novo* pathway requires the two key enzymes serine acetyltransferase and cysteine synthase (Nozaki *et al.*, 2001 ▸). Here, we have analysed the cysteine synthases from the three protozoan pathogens *T. cruzi*, *T. theileri* and *L. infantum*. The three enzymes were recombinantly produced and were biophysically and biochemically analysed in a 96-well plate-assay format optimized for future ligand-screening experiments. Biochemical analysis revealed that all three proteins catalyse the production of cysteine from *O*-acetylserine with high efficiency. Both *Tc*CS and *Tth*CS produce cysteine at a comparable maximum and begin to plateau at a similar concentration of OAS. In comparison, *Li*CS is capable of increased cysteine production and can use a higher concentration of OAS before plateauing. The enzymatic activity is comparable to that found in homologues from other organisms (Bonner *et al.*, 2005 ▸; Westrop *et al.*, 2006 ▸; Mino & Ishikawa, 2003 ▸).

In order to unravel the molecular basis of enzymatic activity and lay the foundation for a structure-based drug-discovery program, high-resolution structures have been obtained of all three protozoan parasite enzymes studied. Both the *Tc*CS and *Tth*CS structures were solved in the closed form with the essential cofactor PLP bound to the active site via a covalent bond to a critical lysine residue. Additionally, the presence of OAS in the active site of *Tc*CS shows a reactive intermediate and represents an additional stage of the reaction process. The structure of *Li*CS was resolved in the open apo form, showing key changes when PLP is not bound to the active site, specifically the absence of a loop covering the active site. Furthermore, a hydrophilic pocket was identified at the dimer interface of *Tc*CS, revealing a potential starting point for the development of protein–protein interaction inhibitors. Interrupting the extensive dimer interface is very likely to expose hydrophobic surfaces, leading to aggregation and hence to catalytically inactive proteins. This discovery will hence enable detailed *in silico* studies towards the discovery of protein–protein inhibitors.

The structures of multiple points along the reaction pathway have been produced, which allow a greater understanding of cysteine synthase from these trypanosomatids and present the potential to find new drugs for the treatment of Chagas disease and leishmaniasis based on these structures. These different structures present unique opportunities to compare the structures and form an initial basis for the structure-based design of novel inhibitors of cysteine synthase. Finally, the availability of well diffracting crystals provides the foundation for *in crystallo* fragment screening to take place for the discovery of new inhibitors.

## Supplementary Material

PDB reference: cysteine synthase from *Leishmania infantum*, 8b9m

PDB reference: from *Trypanosoma theileri*, 8b9w

PDB reference: from *Trypanosoma cruzi*, 8b9y

Supplementary Figures and Table. DOI: 10.1107/S2059798323003613/di5064sup1.pdf

## Figures and Tables

**Table 1:** Statistics for data collection and refinement of *Tc*CS, *Li*CS and *Tth*CS Values in parentheses are for the outer shell.

	*Tc*CS	*Li*CS	*Tth*CS
Data collection			
Beamline	I03, DLS	I24, DLS	I24, DLS
Detector	EIGER2 XE 16M	PILATUS3 6M	PILATUS3 6M
Temperature (K)	100	100	100
Wavelength (Å)	0.9762	0.9762	0.9762
Exposure time (s)	0.05	0.02	0.02
Transmission (%)	100	50	50
Rotation range (°)	360	180	180
Image slices (°)	0.1	0.05	0.05
Resolution range (Å)	54.94–1.80 (1.83–1.80)	87.77–1.75 (1.78–1.75)	76.47–2.75 (2.80–2.75)
Space group	*P*2_1_	*P*2_1_2_1_2_1_	*I*23
*a*, *b*, *c* (Å)	54.9, 66.6, 167.4	48.9, 87.8, 138.0	187.4, 187.4, 187.4
α, β, γ (°)	90, 89.5, 90	90, 90, 90	90, 90, 90
*R*_merge_	0.054 (0.216)	0.099 (0.566)	0.213 (0.774)
*R*_p.i.m._	0.038 (0.160)	0.031 (0.228)	0.029 (0.104)
CC_1/2_	0.996 (0.906)	0.991 (0.805)	0.997 (0.962)
Multiplicity	3.37 (3.15)	11.8 (7.8)	56.3 (57.3)
Completeness (%)	98.27 (96.34)	99.6 (97.5)	100 (100)
〈*I*/σ(*I*)〉	12.93 (1.01)	11.96 (1.02)	23.50 (1.81)
Unique reflections	110250 (5376)	60590 (2908)	28493 (1434)
Refinement			
No. of reflections used	110107	60520	28475
*R*_work_	0.191	0.164	0.148
*R*_free_	0.217	0.205	0.209
Protein atoms	9394	4615	4857
Asymmetric unit contents	2 homodimers	Homodimer	Homodimer
Ligands	4 PLP, 1 OAS, 2 glycerol, 1 ribose	—	2 PLP
R.m.s. deviations			
Bond lengths (Å)	0.01	0.01	0.01
Bond angles (°)	1.51	1.80	2.65
Mean *B* values (Å^2^)			
Protein	26.6	26.4	71.5
Water	35.7	34.5	62.4
Ligands	34.3	35.3	85.0
Ramachandran plot (%)			
Favoured	98.2	97.8	95.4
Allowed	1.8	1.8	4.1
PDB code	8b9y	8b9m	8b9w

**Table 2:** *K*_m_ and *V*_max_ for *Li*CS, *Tth*CS and *Tc*CS calculated from the values in Fig. 3 ▸ using *GraphPad Prism* version 9

	*K*_m_ (m*M*)	*V*_max_ (µ*M* min^−1^)
*Li*CS	3.5	97
*Tth*CS	2.4	50
*Tc*CS	3.1	43

**Table 3:** Residues involved in binding to PLP and OAS in *Tth*CS and *Tc*CS

	*Li*CS	*Tc*CS chain *A*	*Tc*CS chains *B*–*D*	*Tth*CS
Form	Apo	Reaction intermediate	Holo	Holo
Flexible loop	Open, disordered	Open, disordered	Closed, ordered	Closed, ordered
Ligand	—	PLP, OAS	PLP	PLP
Ligand-binding residues	—	OAS: Lys52, Asn83; PLP: Lys52, Thr188, Gly189, Asn83	Lys52, Thr188, Gly189, Thr191, Ser275	Lys52, Thr188, Thr191, Ser275
