# Supplementary material for: Cysteine synthase: multiple structures of a key enzyme in cysteine synthesis and a potential drug target for Chagas disease and leishmaniasis
Source: Acta Crystallogr D Struct Biol. 2023 May 19;79(Pt 6):518–30. doi: 10.1107/S2059798323003613 (PMC10233618; doi:10.1107/S2059798323003613)
Supplement: Supplementary file 1 [file d-79-00518-sup1.pdf]

STRUCTURAL BIOLOGY

Volume 79 (2023)

Supporting information for article:

Cysteine synthase: multiple structures of a key enzyme in cysteine synthesis and a potential drug target for Chagas disease and leishmaniasis

Kate Sowerby, Stefanie Freitag-Pohl, Ana Milena Murillo, Ariel Mariano Silber and Ehmke Pohl

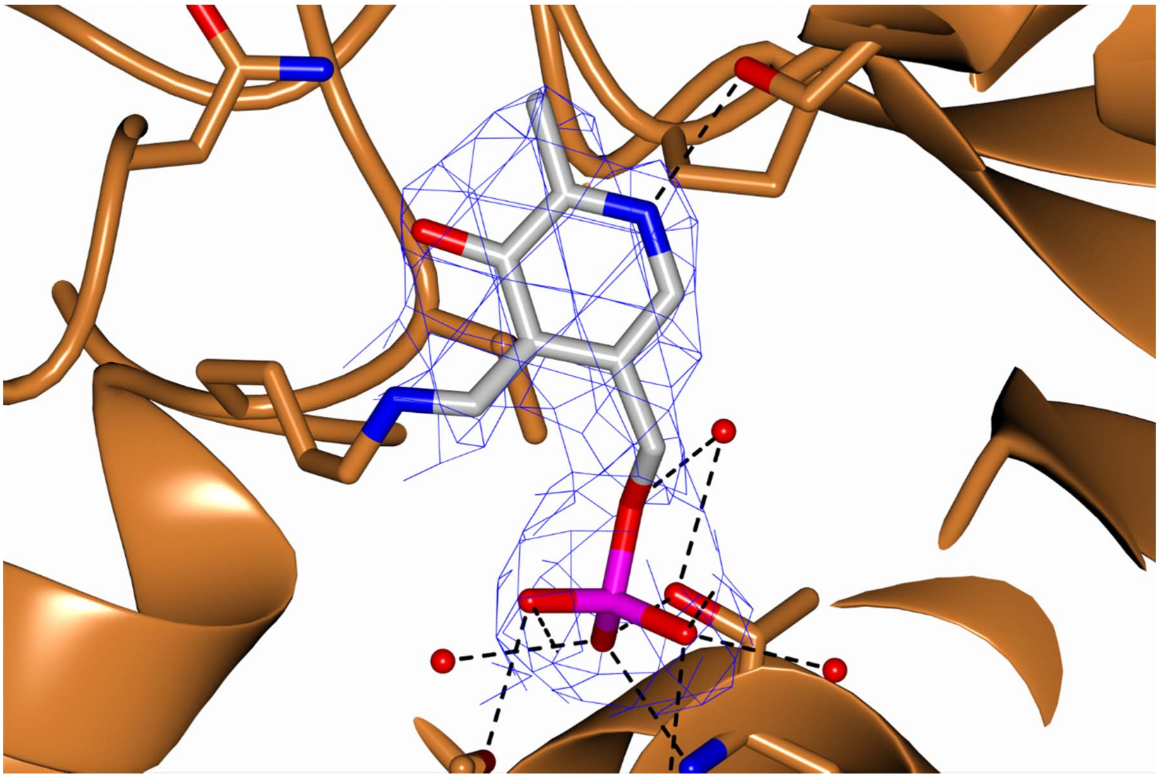

Figure S1 Closeup of the active site of *Tth*CS, showing the protein chain in ribbon representation, the cofactor PLP and the side chains of Lys^{71}, Thr^{207}, Thr^{210} and Ser^{294} in stick representation with N in blue, O in red, C in green and P in magenta. Key hydrogen bonds are indicated by dashed lines. Electron density is shown in blue at 1 σ level in a $2F_o$-F_c map.

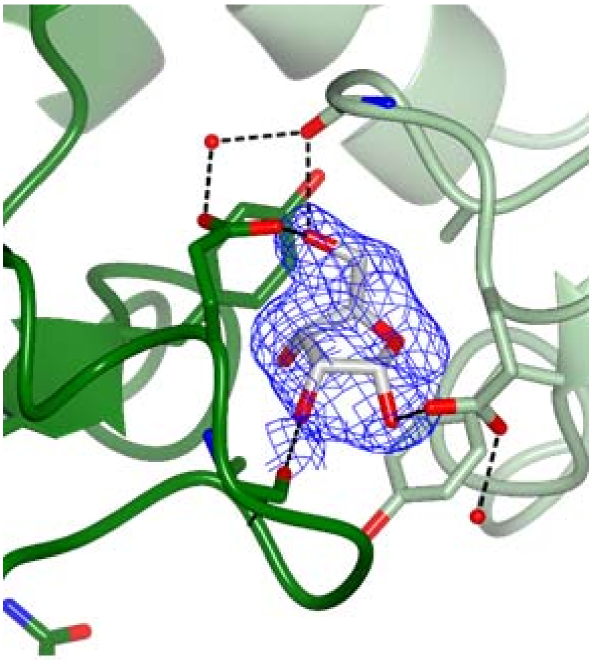

Figure S2 Ribbon diagram of the dimer interface of chain A and B of *Tc*CS – Chain A in dark green, Chain B in light green, ribose shown in stick representation. O is shown in red and N is shown in blue. Residues forming hydrogen bonds and ribose are shown in stick representation with hydrogen bonds displayed as black dashed lines. Electron density is shown in a $2F_o$-F_c map blue at 1 σ level.

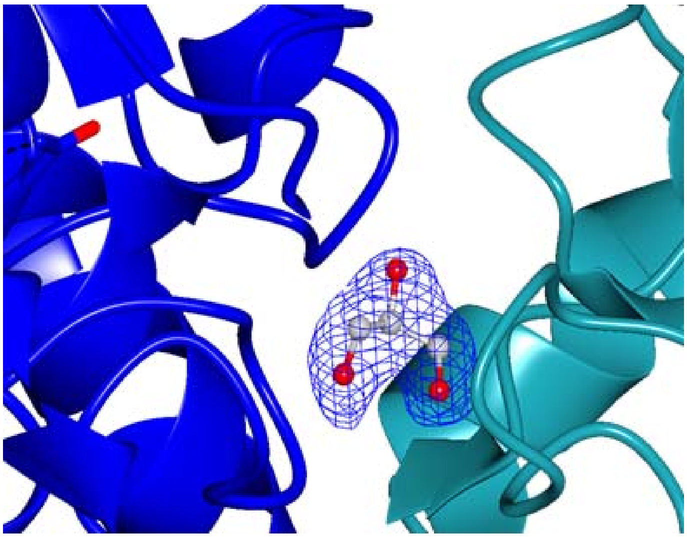

Figure S3 Ribbon diagram of the dimer interface of chain C and D in *Tc*CS - Chain C in blue chain D in dark teal, glycerol is shown in stick representation. O is shown in red. Electron density is shown in a $2F_o$-F_c map blue at 1 σ level.

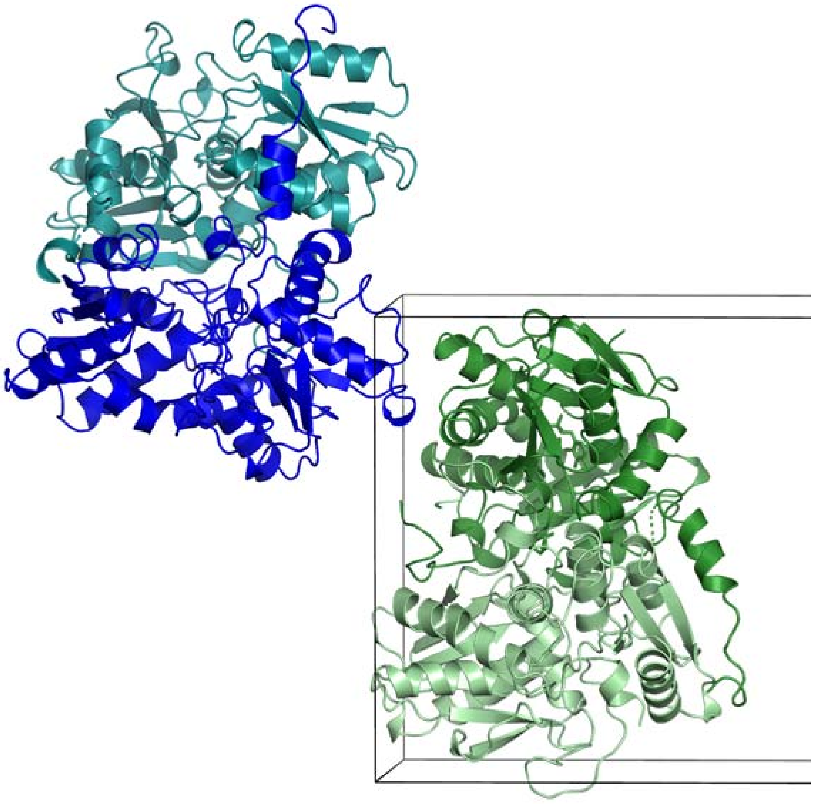

Figure S4 Ribbon diagram of the two crystallographically independent *Tc*CS dimers with the unit cell shown in black: Chain A in dark green, Chain B in light green, Chain C in blue, Chain D in dark teal.

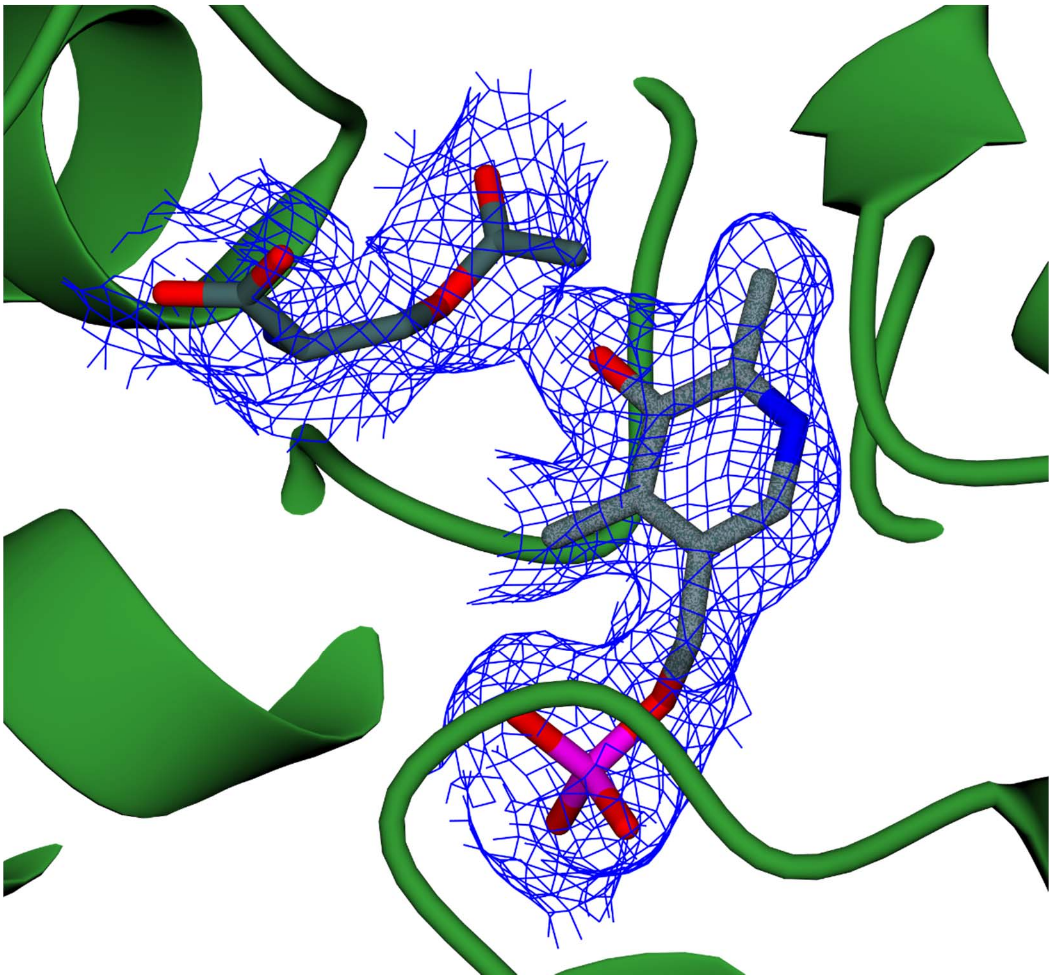

Figure S5 Close up showing active site of *Tc*CS Chain A. Dashed lines indicate hydrogen bonds. Atoms are coloured as before. Electron density is shown in a $2F_o$-F_c map blue at 1 σ level with the electron density indicating the presence of OAS.

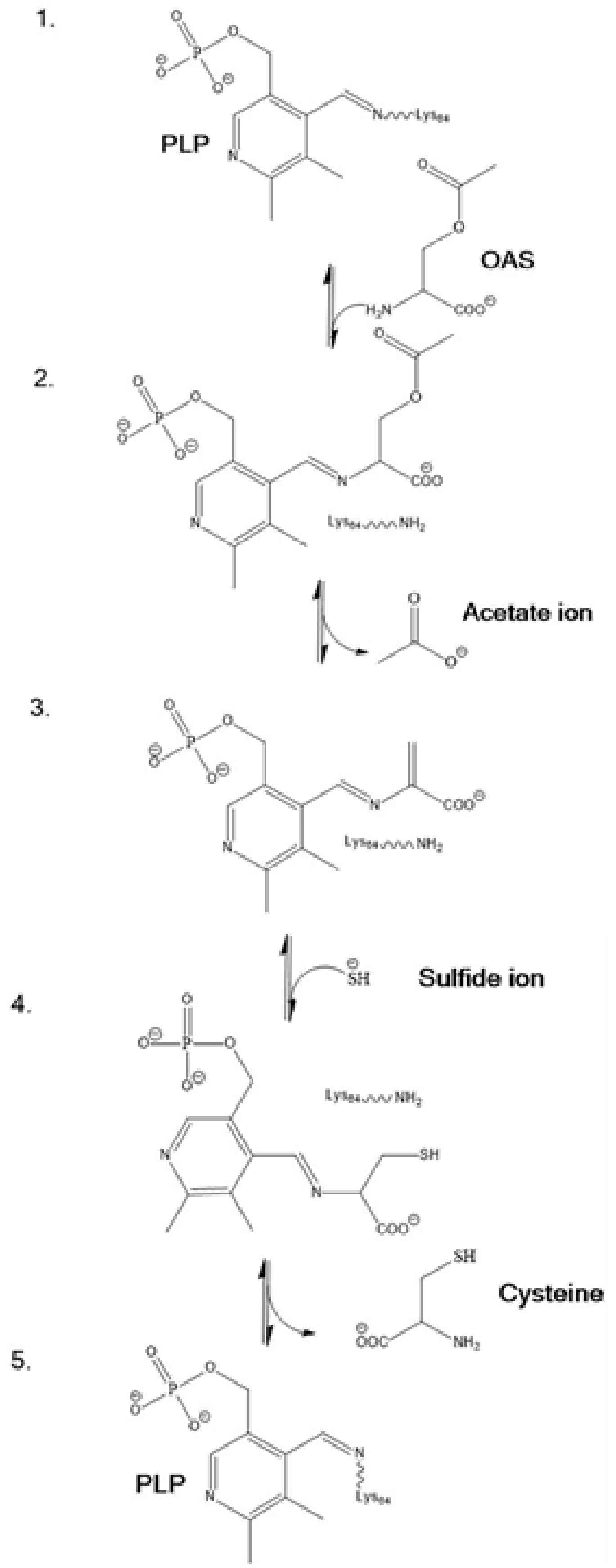

Figure S6 Schema showing reaction of PLP with OAS and a sulfide ion to produce cysteine.

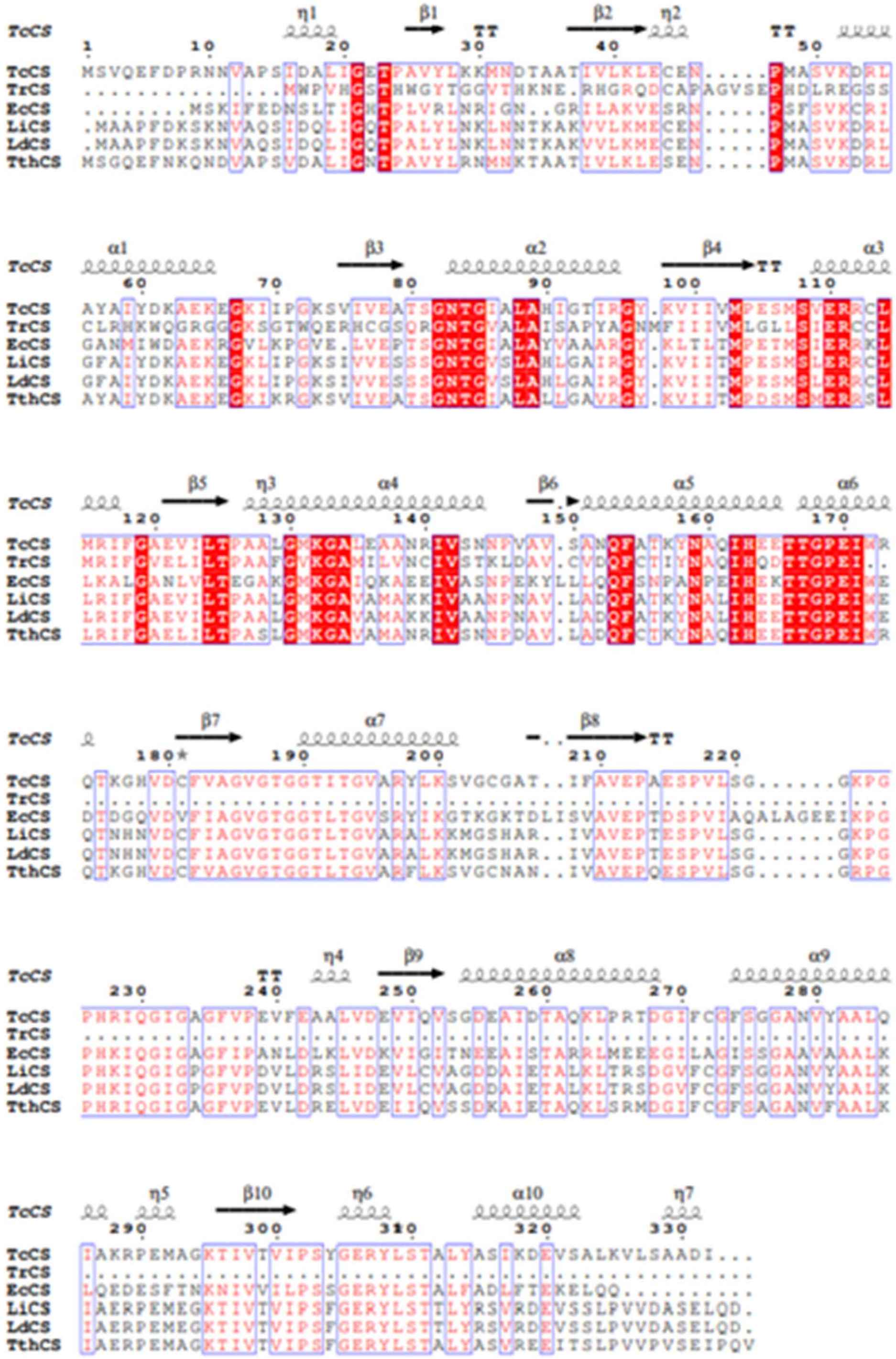

Figure S7 Multiple Alignment of the cysteine synthase sequences of *T. cruzi, T. rangeli, E. coli, L. infantum*, *L. donovani* and *T. theileri*. Identical residues are displayed in a red box, with similar residues in red text, similar or identical residues are framed in a blue box. The secondary structure annotation is based on the *Tc*CS structure presented here.

Table S1 Summary of least-squares superpositions of all protein monomers of the three structures, from *Tc*CS, *Li*CS and *Tth*CS.

Residues 8 – 308 from *Tc*CS and the corresponding residues 5 – 307 from *Li*CS and 6 – 308 from *Tth*CS were used. RMSD values given in Å are presented on the upper right-hand side of table, while the lower left-hand side shows the number of C-alpha atoms used in each least-squares superposition. All calculations were performed with CCP4mg (McNicholas *et al.*, 2011)

	**TcCS A**	**TcCS B**	**TcCS C**	**TcCS D**	**LiCS A**	**LiCS B**	**TthCS A**	**TthCS B**
**TcCS A**		0.54	0.30	0.50	0.70	0.78	0.63	0.56
**TcCS B**	282		0.60	0.17	0.84	1.02	0.64	0.71
**TcCS C**	287	298		0.54	0.78	0.73	0.69	0.63
**TcCS D**	282	303	298		0.83	0.94	0.62	0.68
**LiCS A**	274	270	275	270		0.72	0.74	0.72
**LiCS B**	275	271	274	269	277		1.09	0.97
**TthCS A**	281	303	297	303	268	269		0.74
**TthCS B**	281	303	297	303	269	270	268	
